# Reading speed in school-age children with intermittent exotropia

**DOI:** 10.1038/s41598-022-13293-z

**Published:** 2022-06-08

**Authors:** Cheng Fang, Yidong Wu, Tingting peng, Chunxiao Wang, Jiangtao Lou, Meiping Xu, Jinhua Bao, Chonglin Chen, Xinping Yu

**Affiliations:** 1 Fujian Children’s Hospital, Fuzhou, Fujian China; 2 School of Ophthalmology and Optometry and Eye Hospital of Wenzhou Medical University, Wenzhou, Zhejiang China; 3 Zhongshan Ophthalmic Center of Sun Yat-sen University, Guangzhou, China

**Keywords:** Eye diseases, Ocular motility disorders

## Abstract

Reading speed in intermittent exotropia (IXT) children has been minimally examined. This study assessed reading speed in school-age children with IXT and determined clinical characteristics of IXT that impacted their reading ability. We compared the reading speed of 63 school-age (10–14 years) children with IXT to 44 age-matched normal counterparts. In addition, the correlation between reading speed and clinical characteristics of IXT were evaluated. The reading speed in children with IXT was 231 ± 51 CPM, while reading speed in normal counterparts was 257 ± 33 CPM. Age, gender were found to be factors associated with reading speed in children with IXT. After adjusting for the age and gender, we found a significant correlation between the LogTNO and reading speed in IXT group based on a generalized linear model (p = 0.014). These data show that reading speed was slower in school-age children with IXT assessed with the International Reading Speed Texts. When age and gender were adjusted, poor stereo function at near was found to be related with a slower reading speed.

## Introduction

Intermittent exotropia (IXT) is the most common type of divergent strabismus in children and adolescents^1^. The prevalence of IXT varies in different regions, incidence rates in children are 1% in the United States^2^ and 3–4% in Asia^3,4^. Individuals with IXT are able to maintain ocular alignment and binocular vision for a period of time. However, an abrupt outward deviation of one eye can occur intermittently, thereby disrupting the intact binocular vision^5^. IXT has shown to impact the quality of life for children, including the performance of reading^6,7^, which can be important for a successful academic performance in school-age children.

Previous studies show inconsistent results regarding the reading speed of individuals with strabismus. Some groups showed that amblyopia, not strabismus, is the key factor in slow reading by compare reading speed between strabismic children with amblyopic and strabismic children without amblyopia^8–10^. Others showed that strabismus can directly slow the reading rate in children, who either had an impaired eye movement, poor coordination of binocular saccade coordination or the loss of binocular vision^11–13^.

Studies on reading performance of children with IXT have been relatively minimal. Since the children with IXT could maintain ocular alignment for some period, the effect of IXT on reading is unclear. To illustrate, Hirota et al. used video-oculography to assess the eye movement of eight subjects with convergence insufficiency-type IXT and ten normal observers during reading^14^. They found a significant saccadic disconjugacy in the subjects. Moreover, the subjects often found themselves to be reading the same lines repeatedly, thereby increasing the duration of fixation. However, reading speed was not directly measured in the study. In a subsequent study, Hirota et al. recruited 11 subjects (aged 13–40 years) with IXT and 15 normal observers to study the relationship between binocular coordination and reading performance using video-oculography^15^. They recorded eye movement while the participants read passages from a smartphone at viewing distances of 20, 30 and 50 cm. The proportion of monocular and binocular viewing were also measured from the subjects. The proportion of monocular is exactly the proportion of the exotropia was present. They found that the subjects had a reduced reading speed and a higher proportion of monocular viewing than the normal controls. This phenomenon was more common at a near (i.e. 20 cm) than for viewing distance of 50 cm. In addition, Lions et al. recorded binocular eye movements using an infrared system (mobile EBT) in 18 strabismic children (including 7 IXT subjects) who had been asked to read a four-line text silently (i.e., without verbalizing the text)^16^. They found that the strabismic children had a disrupted binocular saccade coordination, which worsened their reading ability, and a longer duration of fixation during reading. These studies showed that abnormal ocular alignment in IXT subjects could adversely affect the reading performance.

However, the findings of the previous studies on reading performance in IXT cannot be generalized to a large clinical population for several reasons. First, these studies have a small sample size. Second, the differences between previous studies, such as: mix of esotropia and exotropia, combined adults and children,some studies were silent reading, others oral. etc. These differences between studies may resulted in inconsistenct conclusions. Furthermore, for these reasons, the correlation between the reading performance and the clinical characters (i.e. deviation, fusion control, stereoactuity, accommodative and vergence function) in IXT was still remain unclear.

In this study, we aimed to resolve the issues of these previous studies by having a large sample size and employing a standardized reading test to assess reading speed in children with IXT and age-matched normal controls. We used the International Reading Speed Texts (IReST)^17^. IReST contains linguistically standardized passages (around 130–150 words per text). It mimics the level of reading from daily life, and has similar content and linguistic features in 17 different languages. Furthermore, we examined the relationship between the severity of IXT and reading speed of the IXT subjects.

## Subjects and methods

### Participants

63 children with IXT (age range: 10–14 years, including 34 males and 29 females) and 44 cases of age-matched children without strabismus (age range: 10–14 years, including 23 males and 21 females) were enrolled in the study. All subjects gave their assent and a parent gave informed consent.

This prospective case–control study was approved by the ethics committee of the Eye Hospital of Wenzhou Medical University and conformed to the Declaration of Helsinki. All participants were enrolled in the Department of Strabismus from our hospital from June to September 2020. The eligibility criteria were: (1) Definitive diagnosis of IXT, (2) an age range of 10–14 years (at least grade 4), (3) normal vision with a best correct visual acuity (BCVA) better than 20/20, (4) no significant anisometropia, (5) no history of eye surgery or binocular vision training. The exclusion criteria were: (1) anisometropia (spherical equivalent difference ≥ 2.0 diopters), (2) amblyopia (best correct visual acuity < 0.8, or ≥ 2 lines interocular difference by Snellen’s vision chart), (3) binocular vision therapy or extraocular muscle surgery history, (4) convergence insufficiency-type IXT (deviation at near larger than at distance for at least 10 PD), (5) a history of surgery for strabismus or other disease affect reading performance, such as dyslexia, etc. As previous study suggested^18^, we exclude “dyslexia” by asking their history of phonologically based language difficulties (e.g., mispronouncing words, speech punctuated by hesitations and dysfluencies, delayed language, problems with the sounds of words expressive language difficulties, difficulty naming, difficulty learning to associate sounds with letters, history of reading and spelling difficulties in parents and siblings. Futher more,dyslexia not only affect the speed, but also cause kind of errors which can easily find in the reading process.

Age-matched children were enrolled who had normal eye conditions with a possible exception of refractive error.

### Clinical evaluation

The following data were recorded for each patient: gender, age, BCVA, angle of deviation, control of the fusion and the binocular function, including the sensory (sensory fusion, stereoacuity) and motor function (fusional vergence amplitude, amplitude of accommodation and accommodative flexibility). The angle of deviation was measured by a prism and alternate cover test (PACT) at near (1/3 m) and far viewing distances (6 m). An office-based scale was used to assess fusion control in IXT subjects three times (before examination, before and after the reading task) and the average was obtained as the fusional control score, which ranged from 0 to 5. A high control score indicated a poor control of exodeviation^19^. Sensory fusion was evaluated using Worth’s 4-dot test at near and far viewing distances. An abnormal sensory fusion was defined when subjects reported 2 or 3 dots, or 5 dots. A near stereoacuity was assessed using the TNO test (Lameris Intrumenten, Groeningen, Netherlands, 17th Edition). The scores ranged from 15 to 480 s of arc (arcsec). Distance stereoacuity was assessed using the Distance Randot Stereo test (DRS, American Stereo Optical Company); the scores ranged from 63 to 400 arcsec. The scores were transformed into log units for statistical analysis. Stereoacuity was recorded as “nil” if subjects did not pass the test at the largest disparity, and was assigned to the next level of the logarithmic scale, i.e. 960 arc seconds for TNO and 800 arc seconds for RDS^20^.

Fusional vergence amplitudes were measured with a horizontal prism bar while the participants fixated on an accommodative target at near (1/3 m) and far viewing distances (6 m). The base-out prism was gradually increased for a positive vergence (BO) and the base-in prism was gradually increased for a negative vergence (BI). The subjects were asked to identify the point at which the target image appeared to be doubled; this prism power was designated as the breakpoint^21^. BI was first measured before BO to avoid errors caused by the prism requirement of a positive fusion vergence.

#### Near point of convergence (NPC)

Participants were asked to focus on an accommodative target at 40 cm. The target was gradually moved closer to the eyes until they saw double images. The distance between the parallel point of the patient’s lateral canthal and the break point was the NPC.

#### Accommodative flexibility (AF)

Participants were asked to read the "E" visual acuity chart at 40 cm with a ± 2.00 D flipper. During the clinical assessment, the subjects were asked to focus on letters of the chart. After reading the letter successfully, they were asked to turn the flipper and focus on the next letter. This procedure was performed consecutively for 1 minute^22^.

### Reading material

The International Reading Speed Texts (IReST) is a standardized test to assess reading speed and has been proven to be reliable^17,23^. The IReST is printed on white paper at a high contrast, with a letter-size of 10-point Times New Roman font. The level of difficulty is appropriate to children who are10–12 years old and has been linguistically standardized. It was first developed in Europe and has been translated to17 different languages. The Chinese version of IReST consists of 10 essays, each of which contains 153 words. The Chinese version of IReST has been proved useful for assessing reading performance^24^.

### Procedures

The Chinese version of international reading speed texts was performed under the same viewing and luminance conditions for all subjects. The participants were asked to sit comfortably in front of a desk on which the reading card was placed at a distance of 40 cm. Participants were instructed to read each text, loudly and as quickly as possible. Reading speed was measured using a stopwatch that began its count when the participants began to read the first word of each text and stopped when they read the last word. Words read incorrectly or missed were also counted. The reading speed, in character per minute, was calculated using this formula (characters read correctly/seconds) *60 = character count per minute (CPM)^25^. During the training of the reading test, participants were asked to read Text 5 first, then, they received instruction that explained the test procedure. Then, for the actual experiment, they were asked to read out loud Texts 1 and 6. The average reading speed across those from Texts 1 and 6 was then taken as the reading speed of the subjects.

### Data analysis

We grouped all subjects by age, and then calculated the differences in reading speed between IXT and normal children with two-way ANOVA. Another two-way ANOVA was used to calculated the differences in reading speed between male and female in IXT group.A generalized linear model was used to evaluate factors associated with the reading speed in IXT. We select reading speed as the dependent variable,gender as a factor,other parameters such as deviation angle and control score were used as covariables. The results of the generalized linear model suggest that age and gender are related to reading speed. Therefore, we did a partial correlation analysis after controlling for age and gender. A p-value < 0.05 was deemed as statistically significant. All the statistical analysis was performed using SPSS software for Windows version 25.0 (SPSS Inc, Chicago, Illinois, USA). The datasets used and/or analysed during the current study available from the corresponding author on reasonable request.

## Results

### Comparison of binocular functions between children with IXT and normal controls

63 children with IXT and 44 cases of age-matched normal children were enrolled in the study. No significant difference in age and gender between the IXT group and the normal control (Table 1). No significant difference in myopia between IXT group and normal control. Table 1 shows that the IXT group had a poor binocular function than normal control children; this is represented by convergence reserve (BO), negative vergence (BI), sensory fusion, near point of convergence (NPC). Near stereoacuity (LogTNO) and distance stereoacuity LogRDS were worse in IXT than that in normal (p < 0.05).

**Table 1:** Clinical characteristics of 107 subjects.

Parameter	IXT (n = 63)	Control (n = 44)	p value
Age (year,mean(SD))	11.5 ± 1.2	11.2 ± 1.0	0.12
Gender (male:female)	34:29	23:21	0.863
NPC (cm)	5.7 ± 3.6	4.3 ± 1.7	**0.002**
PRA (diopter)	7.2 ± 1.5	6.5 ± 1.7	**0.021**
AF (circle/min)	7.4 ± 3.5	7.1 ± 3.3	0.410
BI@N (prism diopter)	21.7 ± 10.9	15.0 ± 5.2	** < 0.001**
BO@N (prism diopter)	17.0 ± 11.7	28.4 ± 9.69	** < 0.001**
BI@D (prism diopter)	12.4 ± 10.0	11.2 ± 5.5	0.07
BO@D (prism diopter)	8.8 ± 10.1	20.0 ± 11.6	** < 0.001**
Abnormal sensory fusion@N	26.70%	0	
Abnormal sensory fusion@D	63.30%	0	
Myopia	34.9%	31.8%	0.738
LogTNO	2.26 ± 0.44	2.15 ± 0.39	**0.05**
LogRDS	2.36 ± 0.45	2.21 ± 0.47	** < 0.001**
Reading speed in text1 (CPM)	228.7 ± 51.7	254.4 ± 33.6	**0.003**
Reading speed in text6 (CPM)	233.4 ± 53.0	259.1 ± 37.0	**0.008**
Average reading speed (CPM)	231.0 ± 51.2	256.8 ± 33.2	**0.002**

*NPC* near point of convergence, *PRA* positive relative accommodative, *AF* accommodative facility, *BI@N* base in prism power for fusional vergence amplitudes at near, *BO@N* base out prism power for fusional vergence amplitudes at near, *BI@D* base in prism power for fusional vergence amplitudes at distance, *BO@D* base out prism power for fusional vergence amplitudes at distance, *TNO* test for stereoscopic vision, a test for near stereo vision, *RDS* random dot stereo, a test for distance stereo vision, *CPM* character per minute.

Significant values are given in bold.

*p < 0.05 statistically significant.

### Comparison of reading speed between children with IXT and normal controls

**Figure 1:**
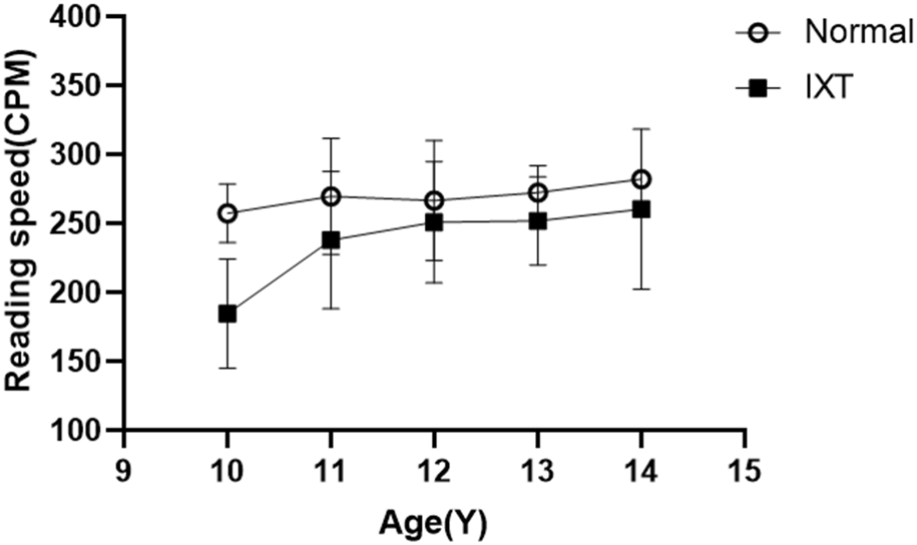
Comparison of reading speed by age between IXT and normal. *CPM* character per minute, *Y* year. Age factor: F = 3.98, p = 0.006 (**); Group factor: F = 21.33, p < 0.001 (***). Reading speed was slower in IXT than normal in each age group.

We found that the average reading speed in the IXT group was 231 ± 51 CPM, whereas that in the normal control group was 257 ± 33 CPM. There was a significant difference between the reading speeds of the two groups (p = 0.002). There was no significant difference in reading speed between Texts 1 and 6, neither in the IXT group (p = 0.865) nor in the control group (p = 0.735). We grouped all subjects by age, and then compared the differences in reading speed between IXT and normal children across age groups by two-way ANOVA. It showed reading speed was slower in IXT than normal children in each age group. Reading speed was correlated with age (F = 3.98, p = 0.006), and more significantly correlated with IXT (F = 21.33, p < 0.001) (Fig. 1). Interestingly, male’s reading speed was significant faster than females in IXT group (p = 0.03), while it was not so in normal control (p = 0.32).

**Figure 2:**
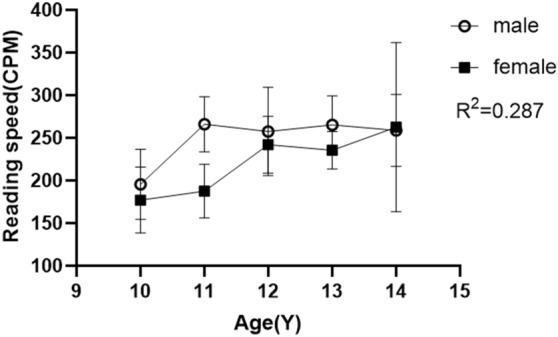
Comparison of reading speed between male and female in IXT. *CPM* character per minute, *Y* year. Age factor: F = 6.29, p = 0.001 (***); Gender factor: F = 6.69, p < 0.017 (**). R^2^ = 0.287, the effect of age and gender on reading speed of IXT was only 28.7%.

We grouped IXT children by age, and compared the differences in reading speed between male and female. It showed the effect of age and gender on reading speed of IXT was only 28.7%, of which age accouted for a larger proportion and gender had a smaller impact (Fig. 2).

### Correlations between reading speed and clinical characteristics in IXT subjects

Correlations between reading speed and clinical factors were analyzed in the IXT subjects. It is commonly believed that stereoacuity is an important factor that describes the severity of IXT. Interestingly, we found that LogTNO was associated with the reading speed using generalized linear model. It implied worse TNO measurement correlated with slower reading speed (Table 2).

**Table 2:** Factors associated with the reading speed in IXT patients.

Parameter	B	Std. error	95% Wald confidence interval		Hypothesis test		
			Lower	Upper	Wald chi-square	df	Sig
(Intercept)	9.482	52.7849	− 93.975	112.939	0.032	1	0.857
Age	19.382	4.8572	9.862	28.902	15.923	1	**0.000**
Gender	33.790	10.2060	13.786	53.793	10.961	1	**0.001**
Deviation angle @N	− 0.098	1.4841	− 3.007	2.811	0.004	1	0.947
Deviation angle @D	0.369	1.5707	− 2.709	3.448	0.055	1	0.814
Control score @N	− 0.953	5.4029	− 11.542	9.637	0.031	1	0.860
Control score @D	− 0.575	4.0786	− 8.569	7.419	0.020	1	0.888
BO@N	− 0.318	0.5161	− 1.329	0.694	0.379	1	0.538
BO@D	− 0.405	0.6626	− 1.704	0.893	0.374	1	0.541
NPC	1.962	1.3816	− 0.746	4.670	2.016	1	0.156
AF	0.837	1.5682	− 2.236	3.911	0.285	1	0.593
LogTNO	− 39.691	16.1554	− 71.355	− 8.027	6.036	1	**0.014**
LogRDS	22.546	16.2451	− 9.293	54.386	1.926	1	0.165
Sensory fusion @N	0.943	13.1229	− 24.777	26.663	0.005	1	0.943
Sensory fusion @D	0.752	12.3699	− 23.493	24.996	0.004	1	0.952

*N* near, *D* distance, *BI* base out prism power for fusional vergence amplitudes, *BO* base out prism power for fusional vergence amplitudes, *NPC* near point of convergence, *AF* accommodative facility, *TNO* test for stereoscopic vision, a test for near stereo vision, *RDS* random dot stereo, a test for distance stereo vision.

Bold mean p < 0.05 statistically significant.

## Discussion

IXT is the most common type of divergent strabismus in children and adolescents, with a prevalence of 3–4% in Asia^3,4^. To our knowledge, our study is the first to examine the reading speed in children with IXT in detail. Reading is a key function in daily life especially for school-age children. Does IXT affect reading performance remain unkown. In our study, we found that children with IXT read more slowly than the age matched normal participants. Moreover, we found worse TNO correlated with slower reading speed. Stereopsis at near might be related with reading speed in children with IXT.

### School-age children with IXT read more slowly than normal

Hirota et al. recruited 11 subjects (aged 13–40 years) with IXT and 15 normal observers to study the relationship between binocular coordination and reading performance using video-oculography^15^. They found that the subjects with IXT had a reduced reading speed and a higher proportion of monocular viewing than the normal controls. Our study, confirmed that school-age children with IXT read more slowly than normal children. The binocular function (e.g. NPC, PRA, Stereopsis etc.) of the IXT group were worse than the normal group, which may be a factor affecting the reading speed.

### Age play a role for reading speed, but not the key factor

In our study, we found that age also play a role for reading speed both in normal and children with IXT. A study in china mainland assessed the reading speed of normally-sighted young Chinese with the same International Reading Speed Texts (IReST) Chinese version which is comprehensible for teenagers and adults. They found the reading speed was 295 CPM in normal adults (18–35 years)^24^. While in our study, the reading speed of the normal children group was significantly lower (257 CPM), which may be related to the younger age (10–14 years). In IXT group, age was also associated with reading speed. But we found the slowing reading speed is mainly caused by IXT, whereas the age account only for a little of the variability. In other words, the presence of IXT, not the age factor, is the key factor in slow reading in school-age children with IXT.

### What correlated with the slow reading speed in children with IXT?

Stereopsis was correlated with reading performance in children with strabismus. Kelly et al. evaluated motor skills in children with strabismus^26^. They found that strabismic children had a poorer stereoacuity and a stronger suppression, both of which could disrupt the development of reading and motor ability. Our results found there was a worse stereopsis in the IXT group compared with the control group. Furthermore, we also showed a negative correlation between reading speed and LogTNO in IXT patients after adjusting for age and gender. Moreover, LogTNO was found as the factors associated with reading speed in the IXT group. This suggests that stereopsis might be related with reading speed in children with IXT.

Previous studies have shown that binocular fusion maintenance (i.e. the vergence function) is related to reading performance^27,28^. Convergence reserve was reported reduced in IXT than in normal observers^29^. Besides, Perrin Fievez et al. and Clotuche et al. found that asymmetric accommodative responses between the dominant eye and the non-dominant eye during binocular viewing in IXT^11,13^. The asymmetric accommodative response was suggested would impact reading performance in subjects with IXT^30^. Our study found that vergence and accommodative functions as well as the steropsis are reduced in individuals with IXT compared with the normal control. While no significant correlation was found between the binocular functions and the reading speed in IXT subjects.

Defining and evaluation the severity of IXT is difficult because there are too many parameters that are seems as associated with the severity. The deviation angle, fusional control score, and stereoacuity are commonly considered as the main factors that affect the severity of IXT^31^. The association between the clinical performance and the severity of the IXT were inconsistent. Some studies showed child HRQOL did not correlate with clinical severity, while others showed a trend toward of HRQOL correlating with clinical severity^32,33^. In our study, no significant correlation was found between the reading speed and the mentioned characters of clinical “severity”. More subjects and wider distribute of clinical parameters, should be included in the future studies, as well as the more precise evaluation of the clinical severity,such as the inter-coualr suppression, the ocular motor parameters and the visual processing. Reading performance is important for children’s academic performance in school^6,7^. Although our findings indicate that the severity of IXT is not correlated with reading speed, we still show that children with IXT have a slower reading speed. Therefore, it might be important to assess reading speed as part of routine clinical examination in children with IXT.

Our study has a few limitations. First, reading speed alone cannot fully reflect reading performance because one can read quickly without comprehension^9^. Second, we asked the participants to read out loud. The children’s speaking rate could differ from the speed of reading comprehension; extracting meaning from a text could be achieved without verbalizing the text. Furthermore, we used the same reading distance for all the subjects, as well as the same distance for assessing the clinical characteristics (i.e., stereoacuity). This study design of using the same distance for all subjects enabled us to easily compare the relationship between reading speed and clinical characteristics^15^. Lastly, saccadic and fixation function have been shown to be associated with reading performance. Disconjugacy saccadic and longer fixation have been found in children with strabismus because the subjects often found themselves rereading the same passage to achieve comprehension. The role of fixation and saccadic parameters in reading performance for subjects with IXT should be investigated in the future by using an eye-tracking device.

In conclusion, reading speed seems to be slower in school-age children with IXT based on the International Reading Speed Texts. Stereoacuity at near was associated with reading speed in children with IXT (Supplementary Information).

## Supplementary Information

Supplementary Information.
